# Polydeoxyribonucleotide Mitigates Dextran Sulfate Sodium-Induced Ulcerative Colitis in Mice Through the PKA/CREB/NF-κB Signaling Pathway

**DOI:** 10.3390/biomedicines14061241

**Published:** 2026-05-29

**Authors:** SeungHwan Lee, Lakkyong Hwang, Sang Hoon Kim, Sang Hoon Lee, Jin Hee Han, Jung Won Jeon, Hyeong Chan Shin, Il-Gyu Ko

**Affiliations:** 1 Department of Surgery, Kyung Hee University Hospital at Gangdong, College of Medicine, Kyung Hee University, Seoul 05278, Republic of Korea; histones@hanmail.net; 2 Team of Efficacy Evaluation, Orient Genia Inc., Seongnam-si 13201, Republic of Korea; lhwangphd@gmail.com; 3 Department of Neurosurgery, Robert Wood Johnson Medical School Rutgers, The Stat University of New Jersey, Piscataway, NJ 08854, USA; sanghoon.kim1@rutgers.edu (S.H.K.); sl2390@rwjms.rutgers.edu (S.H.L.); 4 Department of Anesthesiology and Pain Medicine, Kyung Hee Medical Center, College of Medicine, Kyung Hee University, Seoul 02447, Republic of Korea; esthesi@khu.ac.kr; 5 Department of Internal Medicine, Kyung Hee University Hospital at Gangdong, College of Medicine, Kyung Hee University, Seoul 05278, Republic of Korea; drglory@naver.com; 6 Department of Pathology, School of Medicine, Keimyung University, Daegu 42601, Republic of Korea; chan@dsmc.or.kr; 7 Research Support Center, School of Medicine, Keimyung University, Daegu 42601, Republic of Korea

**Keywords:** ulcerative colitis, polydeoxyribonucleotide, adenosine A_2A_ receptor, inflammation, angiogenesis

## Abstract

**Background/Objectives:** Ulcerative colitis (UC) is a type of inflammatory bowel disease characterized by abdominal pain, diarrhea, and bleeding. Polydeoxyribonucleotide (PDRN), an adenosine A_2A_ receptor (A_2A_R) agonist, exhibits anti-inflammatory properties. In the present study, we evaluated the therapeutic effects of PDRN in a dextran sodium sulfate (DSS)-induced murine model of UC. **Methods:** UC was induced by administering 2% DSS in drinking water for 7 days. One day after DSS administration, mice received intraperitoneal injections of PDRN (8 mg/kg) for 7 days. To investigate the involvement of A_2A_R, the selective antagonist 3,7-dimethyl-1-propargylxanthine (DMPX, 8 mg/kg) was co-administered with PDRN. **Results:** DSS administration induced colonic tissue damage and increased disease activity index (DAI) and histological scores. DSS also elevated pro-inflammatory cytokines while reducing anti-inflammatory cytokine levels. PDRN treatment reduced histological damage, restored body weight, colon weight, and colon length, and decreased DAI scores. Furthermore, PDRN treatment inhibited nuclear factor kappa B (NF-κB) activation through suppression of NF-κB inhibitor-α phosphorylation and was associated with activation of the cAMP/PKA/CREB signaling pathway. PDRN treatment attenuated inflammation and was associated with increased expression of vascular endothelial growth factor (VEGF) in colonic tissues. Given the context-dependent role of VEGF in inflammatory bowel disease, this increase is interpreted as contributing to mucosal repair rather than exacerbating inflammation. Co-administration of DMPX abolished these effects, suggesting the involvement of A_2A_R-dependent signaling pathways. **Conclusions:** PDRN attenuated colonic inflammation and improved disease outcomes in DSS-induced UC, potentially through modulation of the PKA/CREB/NF-κB signaling pathway and VEGF-mediated tissue repair mechanisms.

## 1. Introduction

Ulcerative colitis (UC) is a type of inflammatory bowel disease whose main symptoms are abdominal pain, diarrhea, and bleeding. UC affects the quality of life of patients, leading to long-term and debilitating complications [1]. The underlying mechanisms of UC are complex, but inflammation is a key factor in its pathogenesis [2]. When gut microbes invade the lamina propria of the intestinal mucosa, macrophages are polarized to the M1 inflammatory phenotype, secrete many pro-inflammatory cytokines and chemokines, and recruit a lot of immune cells to infiltrate the intestinal and colonic tissues [3]. Excessive immune responses can cause extensive epithelial cell damage, leading to tissue repair imbalance, which may lead to UC [4].

Nuclear factor kappa B (NF-κB), a key factor of the cell signaling system, regulates immune response and inflammation and is closely implicated in the pathogenesis of UC [5,6]. Various inflammatory factors depend on NF-κB-related signaling, and when exposed to extracellular stimuli, inhibitors of NF-κB (IκB) are rapidly phosphorylated and degraded, which is mediated by IκB kinase [7]. The NF-κB/IκB complex has been suggested to be closely related to UC progression [8,9].

Angiogenesis is important for wound healing, including colonic mucosal healing [10]. Among many angiogenic factors, vascular endothelial growth factor (VEGF) is one of the most potent stimulators of angiogenesis and acts as a key factor in colonic mucosal healing [11]. Since UC is characterized by recurrent inflammation, rapid tissue regeneration at the damaged site is essential for effective treatment. An adenosine A_2A_ receptor (A_2A_R) agonist, polydeoxyribonucleotide (PDRN), has an anti-inflammatory effect by inhibiting the production of pro-inflammatory cytokines. Pallio et al. [12] demonstrated that PDRN treatment reduced the level of inflammatory factors in experimental colitis rats. PDRN treatment alleviated symptoms of various inflammatory diseases through its anti-inflammatory and anti-apoptotic properties [13,14].

Increasing VEGF production, suppression of inflammation, and inhibition of apoptosis are considered important therapeutic strategies for UC. Angiogenesis and inflammatory regulation are closely associated with mucosal healing and disease progression in UC. Therefore, we investigated the therapeutic effect of PDRN in a DSS-induced UC model by examining its effects on the A_2A_R-mediated PKA/CREB/NF-κB signaling pathway and VEGF expression. Through this mechanistic approach, we aimed to better understand the role of PDRN in UC and its potential therapeutic relevance in inflammatory bowel disease.

## 2. Materials and Methods

### 2.1. Experimental Animals and Grouping

Forty adult male C57BL/6N mice, aged 11 weeks and weighing 30 ± 2 g, were sourced from Orient Bio Co. (Sungnam, Republic of Korea). After acclimatization, the mice were randomly allocated into experimental groups using a simple randomization method. These mice were assigned to one of four experimental groups (n = 10 per group): control group, dextran sulfate sodium (DSS)-treated group, DSS and PDRN-treated group, and DSS and PDRN with 3,7-dimethyl-1-propargylxanthine (DMPX)-treated group. Additional animals were initially included in the DSS-treated groups, considering possible loss during the experimental period associated with DSS-induced colitis, and the final analysis was performed using 10 animals per group.

All animal experimental procedures were approved by the Institutional Animal Care and Use Committee (IACUC) of Kyung Hee University (Approval No. KHUASP-20-662, Approval Date: 23 December 2020), where the experiments were conducted, and were performed in accordance with the National Institutes of Health Guide for the Care and Use of Laboratory Animals.

### 2.2. Induction of UC and Treatment

In this study, UC was created by administering DSS according to a previously established protocol [15,16]. This UC mouse model was induced by supplementing drinking water with 2% DSS (MP Bio, Santa Ana, CA, USA) for 7 days, after which the water was switched to regular drinking water for an additional 8 days before the animals were sacrificed. 

**Figure 1:**
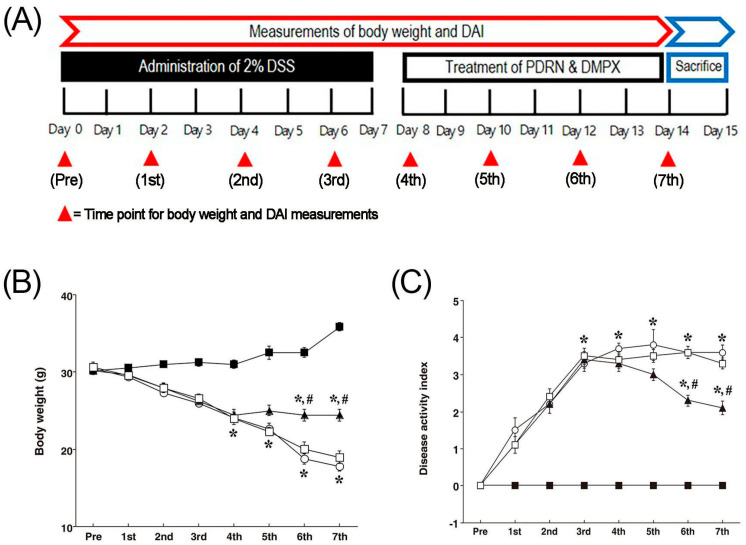
Alterations of body weight and disease activity index score. (**A**) Experimental schedule. (**B**) Body weight. (**C**) Disease activity index score. ■, control group; ○, dextran sulfate sodium-treated group; ▲, dextran sulfate sodium and polydeoxyribonucleotide-treated group; □, dextran sulfate sodium and polydeoxyribonucleotide with 3,7-dimethyl-1-propargylxanthine-treated group. * represents *p* < 0.05 compared to the control group. # represents *p* < 0.05 compared to the dextran sulfate sodium administration group.

Drug treatments were initiated one day after DSS administration. Mice in the PDRN-treated groups received intraperitoneal injection of 8 mg/kg PDRN (Rejuvenex^®^, PharmaResearch Products, Pangyo, Republic of Korea) in 200 μL normal saline once a day for 7 days. To investigate the role of A_2A_R in the effects of PDRN, 8 mg/kg of the A_2A_R antagonist DMPX (Sigma Chemical Co., St. Louis, MO, USA) was administered together with PDRN. The experimental timeline is depicted in Figure 1A. The company had no role in study design, data collection, data analysis, interpretation of the results, or manuscript preparation.

### 2.3. Measurements of Disease Activity Index (DAI), Colon Wet Weight, and Colon Length

To observe DAI, scores were calculated by the criteria explained in [17,18]. DAI was measured every 2 days from day 0 to day 15, monitoring body weight loss, stool viscosity, and rectal bleeding (total 7 times). The total score is the sum of the individual grades of the three indices above. All animals were euthanized, and the same part of the distal colon was collected. The colonic segments were photographed at the same angle and distance to analyze morphological changes and colon length, and the colonic wet weight was measured.

### 2.4. Tissue Preparation and Hematoxylin and Eosin Staining

Colon tissues were fixed using a 4% paraformaldehyde solution, embedded in paraffin, and then thin sections (5 μm thick) were stained with hematoxylin and eosin in accordance with the standard protocol described in [19,20]. UC damage score was calculated according to the Wallace histological colon damage scoring system (Table 1). This assessment was conducted in a double-blind manner, and two independent pathologists interpreted the results to prevent bias.

### 2.5. Measurement of Pro- and Anti-Inflammatory Cytokines, cAMP, and A_2A_R Level

The concentrations of pro-inflammatory cytokines, including necrosis factor (TNF)-α, interleukin (IL)-1β, and IL-6, as well as anti-inflammatory cytokines, such as IL-4 and IL-10, and cyclic adenosine monophosphate (cAMP) in colonic tissues, were measured using enzyme-linked immunosorbent assay (ELISA) kits, following the manufacturer’s protocols (Abcam, Cambridge, UK). The level of A_2A_R was determined using a specific ELISA kit obtained from MyBioSource (San Diego, CA, USA), according to the supplier’s instructions.

### 2.6. Western Blotting

Western blotting was conducted as per the method in [20]. Colonic tissues were homogenized using cold radioimmunoprecipitation assay buffer (Cell Signaling Technology Inc., Danvers, MA, USA) containing 1 mM phenylmethylsulfonyl fluoride (Sigma Aldrich Co., St. Louis, MO, USA). The homogenized material was centrifuged at 14,000 rpm for 30 min at 4 °C. Subsequently, 30 μg of protein was separated on a sodium dodecyl sulfate-polyacrylamide gel and transferred to a nitrocellulose membrane. The membrane was incubated with primary antibodies against β-actin (1:1000; Santa Cruz Biotechnology, Santa Cruz, CA, USA) and NF-κB, IκB-α, p-IκB-α, CREB, p-CREB, PKA, and p-PKA (1:1000; Cell Signaling Technology, Danvers, MA, USA). After incubation with horseradish peroxidase-conjugated secondary antibodies (1:2000; Vector Laboratories, Burlingame, CA, USA), immunoreactive bands were detected using an enhanced chemiluminescence detection kit (Bio-Rad, Hercules, CA, USA). Bands were analyzed densitometrically with an Image-Pro^®^ Plus computer-assisted image analysis system (Media Cybernetics Inc., Silver Spring, MD, USA) for the relative protein expression levels. The value of the control group was set at 1.00.

### 2.7. Immunofluorescence Staining of VEGF

Immunofluorescence staining for VEGF in the colonic tissues was performed as per the method in [21]. Paraffin-embedded sections with colonic tissues were deparaffinized by xylene, fractionated in ethanol for 5 min, and washed with distilled water for 5 min. Slides were placed in 10 mM sodium citrate buffer, boiled at 95 °C for 2 min, and cooled at room temperature for 30 min. Slides were blocked by phosphate-buffered saline with 5% normal goat serum (Vector Laboratories, Burlingame, CA, USA). Slides were treated overnight at 4 °C with a 1:100 dilution of mouse anti-VEGF antibody (Santacruz Biotechnology, Dallas, TX, USA). 

After incubation with the primary antibody, the slides were incubated with secondary fluorescein isothiocyanate (FITC) anti-mouse IgG (Jackson ImmunoResearch Laboratories, West Grove, PA, USA). The slides were mounted on the coverslips using Vectashield^®^ containing 4′,6-diamidino-2-phenylindole. Images were acquired using a Leica DMi8 fluorescence microscope (Leica Microsystems, Wetzlar, Germany) at an excitation wavelength of 490 nm and an emission wavelength of 525 nm for FITC.

### 2.8. Data Analysis

Statistical analyses were performed using SPSS software (version 23.0; IBM Corporation, Armonk, NY, USA). Normality of continuous variables was confirmed prior to parametric analysis. Repeated measurements of body weight and disease activity index were analyzed using two-way repeated measures ANOVA. For single-endpoint data, one-way ANOVA followed by Duncan’s post hoc test was used for comparisons among groups. Histological scoring data were analyzed using non-parametric methods. Data are expressed as mean ± standard error of the mean. Statistical significance was defined as *p* < 0.05. All experimental procedures and data analyses were performed under blinded conditions using coded group assignments.

## 3. Results

### 3.1. Effect of PDRN Treatment on Body Weight and DAI Score

The change in body weight in DSS-induced UC mice is shown in Figure 1B. DSS administration resulted in a gradual decrease in body weight compared to the control group (*p* < 0.05), and this reduction persisted throughout the experimental period. During the early phase of DSS administration (Pre to 4th time point), no significant differences were observed between the DSS and PDRN-treated groups. Following the initiation of PDRN treatment (from the 4th time point), attenuation of body weight loss became evident, and body weight was significantly increased compared to the DSS group from the 5th time point onward (*p* < 0.05). This protective effect was maintained until the end of the experiment. In contrast, co-treatment with DMPX attenuated the beneficial effect of PDRN on body weight.

Parallel changes were observed in the DAI score (Figure 1C). DSS-treated mice showed a marked increase in DAI score compared to control mice as early as the 1st–2nd time points, and these elevated levels were sustained throughout the experimental period (*p* < 0.05). During the early phase (Pre to 4th time point), no significant differences were observed between the DSS and PDRN-treated groups. However, after the initiation of PDRN treatment, DAI scores were significantly reduced compared to the DSS group from the 5th time point onward (*p* < 0.05), and this effect persisted until the end of the experiment. Co-administration of DMPX partially reversed the inhibitory effect of PDRN on DAI score, suggesting that the therapeutic effect of PDRN is mediated through A_2A_R.

### 3.2. Effect of PDRN Treatment on Colon Length and Wet Weight

**Figure 2:**
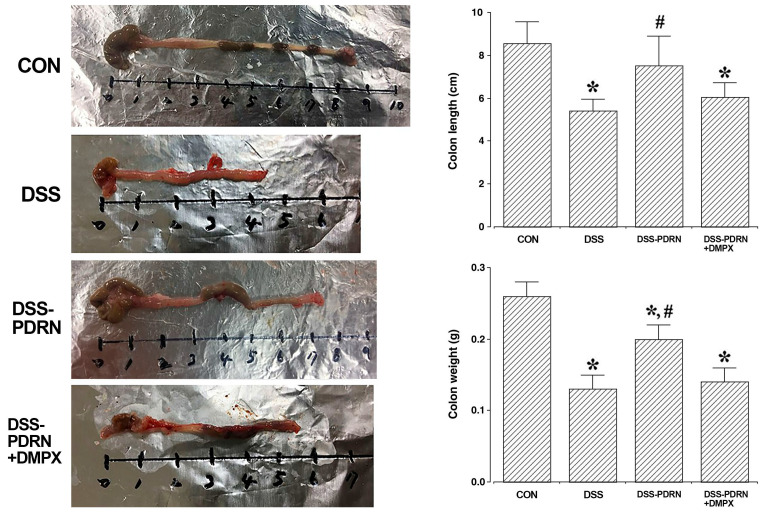
Alteration of colon length and weight. Left: Representative photo of colon tissues. Right upper: Change in colon length. Right lower: Change in colon weight. (CON) control group; (DSS) dextran sulfate sodium-treated group; (DSS-PDRN) dextran sulfate sodium and polydeoxyribonucleotide-treated group; (DSS-PDRN + DMPX) dextran sulfate sodium and polydeoxyribonucleotide with 3,7-dimethyl-1-propargylxanthine-treated group. * represents *p* < 0.05 compared to the control group. # represents *p* < 0.05 compared to the dextran sulfate sodium administration group.

The change in colon length and weight in the DSS-induced UC mice is shown in Figure 2. DSS-induced UC mice exhibited a significant reduction in both colon length and wet weight compared to control mice (*p* < 0.05), reflecting inflammation-associated tissue damage and shrinkage. However, PDRN treatment significantly restored colon length and wet weight in UC mice (*p* < 0.05), indicating a protective effect against colonic atrophy. In contrast, co-treatment with the A_2A_R antagonist DMPX attenuated these restorative effects, as colon length and weight remained reduced despite PDRN treatment (*p* < 0.05). These findings may suggest that the beneficial effects of PDRN on colonic structure are mediated through A_2A_R-dependent signaling pathways.

### 3.3. Effect of PDRN Treatment on Histological Evaluation

**Figure 3:**
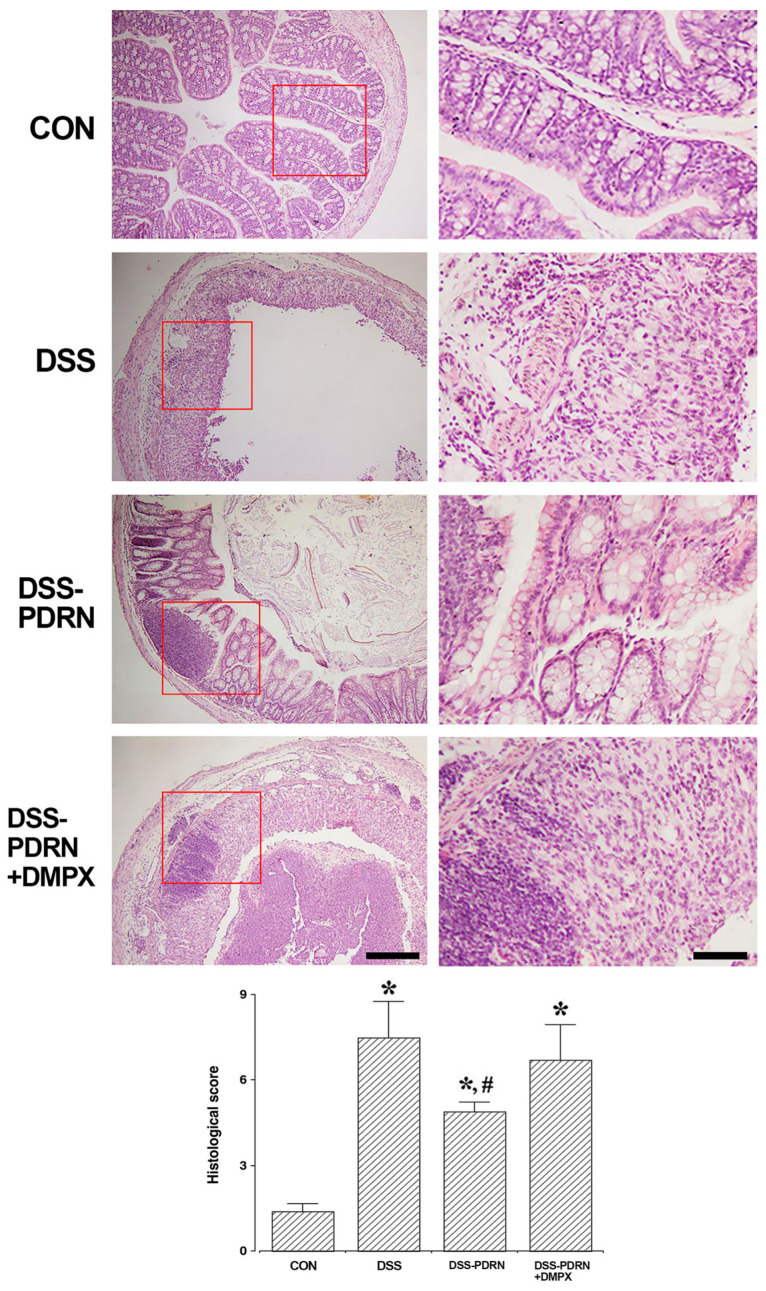
Alterations of histological characteristics and histological damage score. Upper: The sections were stained with hematoxylin and eosin (H&E), in which nuclei appear dark purple and cytoplasmic structures appear light purple to pink. The scale bars represent 50 μm (right photo) and 150 μm (left photo). Lower: Change in histological score. (CON) control group; (DSS) dextran sulfate sodium-treated group; (DSS-PDRN) dextran sulfate sodium and polydeoxyribonucleotide-treated group; (DSS-PDRN + DMPX) dextran sulfate sodium and polydeoxyribonucleotide with 3,7-dimethyl-1-propargylxanthine-treated group. * represents *p* < 0.05 compared to the control group. # represents *p* < 0.05 compared to the dextran sulfate sodium administration group.

Figure 3 shows the histological characteristics in the colonic tissues of the DSS-induced UC mice. The colonic mucosa of the control mice had an intact epithelium and a normal appearance, whereas the mucosa of UC mice had mucosal damage, loss of goblet cells, abnormal papillae, and infiltration of inflammatory cells. These changes led to an increment in the colon damage score (*p* < 0.05). However, PDRN treatment markedly improved mucosal integrity, reduced inflammatory infiltration, and restored crypt structure, leading to a significant decrease in the colon damage score (*p* < 0.05). 

In contrast, when PDRN was co-administered with the A_2A_R antagonist DMPX, these histological improvements were markedly reduced, and the damage score remained elevated (*p* < 0.05). These findings indicate that PDRN exerts mucosal protective and regenerative effects in colonic tissue via A_2A_R-mediated pathways.

### 3.4. Effect of PDRN Treatment on Pro- and Anti-Inflammatory Cytokines

**Figure 4:**
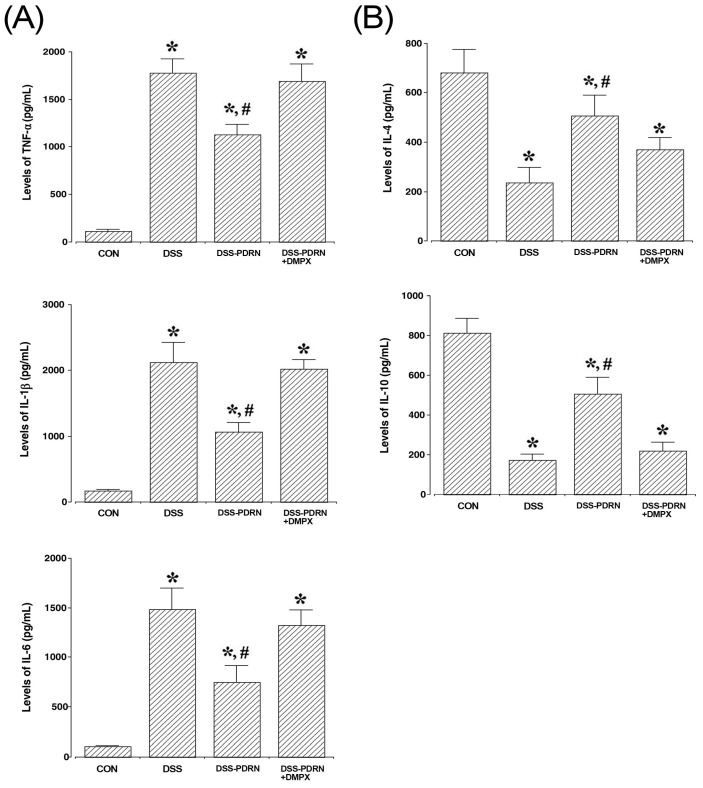
Alterations of pro-inflammatory cytokines, such as tumor necrosis factor (TNF)-α, interleukin (IL)-1β, and IL-6, and anti-inflammatory cytokines, such as IL-4 and IL-10 expression. (**A**) Expression of TNF-α, IL-1β, and IL-6 in colon tissues. (**B**) Expression of IL-4 and IL-10 in colon tissues. (CON) control group; (DSS) dextran sulfate sodium-treated group; (DSS-PDRN) dextran sulfate sodium and polydeoxyribonucleotide-treated group; (DSS-PDRN + DMPX) dextran sulfate sodium and polydeoxyribonucleotide with 3,7-dimethyl-1-propargylxanthine-treated group. * represents *p* < 0.05 compared to the control group. # represents *p* < 0.05 compared to the dextran sulfate sodium administration group.

The expression levels of pro-inflammatory cytokines such as TNF-α, IL-1β, and IL-6 and anti-inflammatory cytokines such as IL-4 and IL-10 in the colonic tissues of the DSS-induced mice are shown in Figure 4A,B. UC induction enhanced the expression level of pro-inflammatory cytokines (*p* < 0.05), while the expression level of anti-inflammatory cytokines was decreased in UC mice (*p* < 0.05). However, PDRN treatment reversed these cytokine imbalances by suppressing pro-inflammatory cytokine (*p* < 0.05) expression and enhancing anti-inflammatory cytokine levels in colonic tissue in UC mice (*p* < 0.05). 

When PDRN and DMPX were co-treated, the suppressive effects of PDRN on pro-inflammatory cytokines and the stimulatory effects of PDRN on anti-inflammatory cytokines were significantly attenuated (*p* < 0.05).

This shows that these effects of PDRN on pro-inflammatory cytokines, anti-inflammatory cytokines, and cAMP were mediated through A_2A_R.

### 3.5. Effect of PDRN Treatment on cAMP and A_2A_R Expression

**Figure 5:**
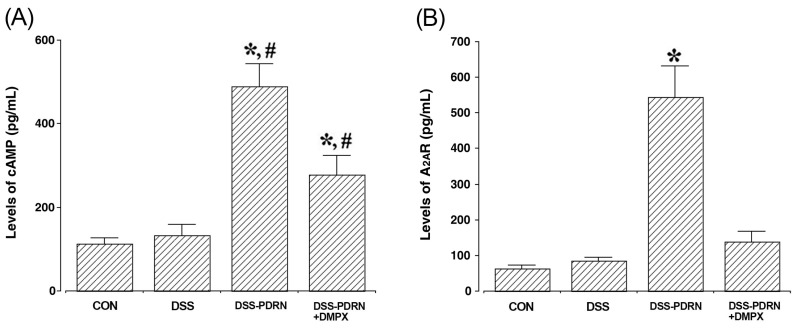
Alterations of cyclic adenosine monophosphate (cAMP) and adenosine A_2A_ receptor (A_2A_R) expression. (**A**) Expression of cAMP in colon tissues. (**B**) Expression of A_2A_R in colon tissues. (CON) control group; (DSS) dextran sulfate sodium-treated group; (DSS-PDRN) dextran sulfate sodium and polydeoxyribonucleotide-treated group; (DSS-PDRN + DMPX) dextran sulfate sodium and polydeoxyribonucleotide with 3,7-dimethyl-1-propargylxanthine-treated group. * represents *p* < 0.05 compared to the control group. # represents *p* < 0.05 compared to the dextran sulfate sodium administration group.

The levels of cAMP and A_2A_R expression in the colonic tissues of the DSS-induced mice are shown in Figure 5A,B. DSS administration resulted in a slight increase in both cAMP and A_2A_R expression in UC mice compared to control mice. However, these changes were not statistically significant. In contrast, following PDRN treatment, both cAMP and A_2A_R expression were significantly enhanced in the colonic tissue of DSS-induced UC mice (*p* < 0.05), indicating activation of A_2A_R-mediated signaling pathways.

Importantly, the co-administration of PDRN with the DMPX significantly attenuated these effects, with cAMP and A_2A_R expression reduced compared to the PDRN-treated group (*p* < 0.05). These findings further support that actions of PDRN are mediated via A_2A_R activation.

### 3.6. Effect of PDRN Treatment on PKA/CREB/NF-κB Signaling Pathway

**Figure 6:**
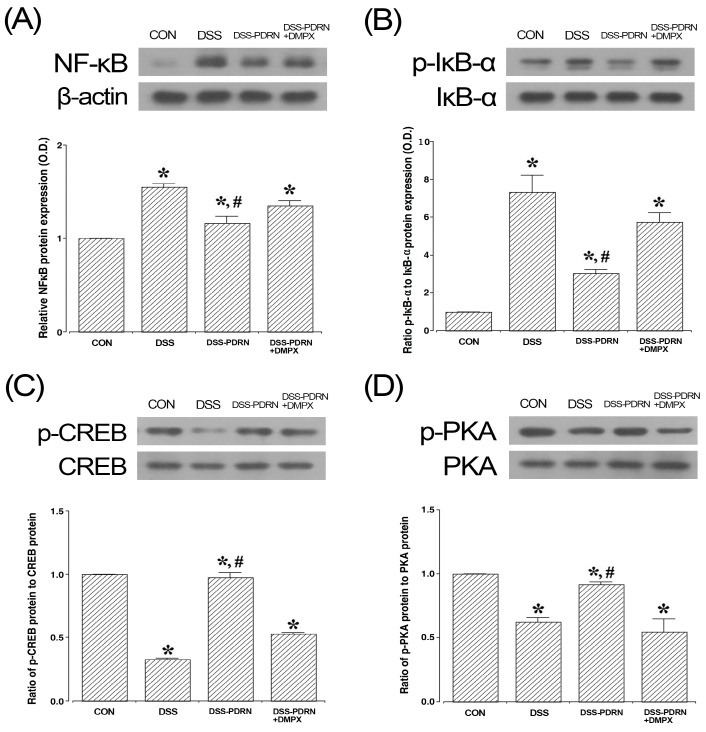
Alterations of nuclear factor-κB (NF-κB), NF-κB inhibitor-α (IκB-α), cAMP response element-binding protein (CREB), and protein kinase A (PKA) expression. (**A**) The relative NF-κB expression. Actin was used as an internal control (46 kDa). (**B**) The relative ratio of phosphorylated (p)-IκB-α to IκB-α expression. (**C**) The relative ratio of p-CREB-α to CREB expression. (**D**) The relative ratio of p-PKA to PKA expression. (CON) control group; (DSS) dextran sulfate sodium-treated group; (DSS-PDRN) dextran sulfate sodium and polydeoxyribonucleotide-treated group; (DSS-PDRN + DMPX) dextran sulfate sodium and polydeoxyribonucleotide with 3,7-dimethyl-1-propargylxanthine-treated group. * represents *p* < 0.05 compared to the control group. # represents *p* < 0.05 compared to the dextran sulfate sodium administration group.

NF-κB activation and PKA/CREB phosphorylation in the colonic tissues of the DSS-induced UC mice are shown in Figure 6. DSS administration induced the phosphorylation of NF-κB inhibitor-α (IκB-α), which increased NF-κB expression (*p* < 0.05), indicating activation of NF-κB. However, treatment with PDRN suppressed IκB-α phosphorylation and NF-κB expression in UC mice (*p* < 0.05). Furthermore, induction of UC using DSS administration suppressed the ratio of *p*-CREB/CREB and *p*-PKA/PKA compared to those in the control group (*p* < 0.05). However, treatment with PDRN enhanced the ratio of p-CREB/CREB and p-PKA/PKA in UC mice (*p* < 0.05). 

When PDRN and DMPX were co-treated, the decreasing effect of PDRN on IκB-α phosphorylation and NF-κB expression and the enhancing effect of PDRN on PKA and CREB phosphorylation disappeared after co-treatment with DMPX (*p* < 0.05). These findings indicate that the regulatory effects of PDRN on the PKA/CREB/NF-κB signaling pathway are mediated through A_2A_R activation.

### 3.7. Effect of PDRN Treatment on VEGF-Positive Cell Expression

**Figure 7:**
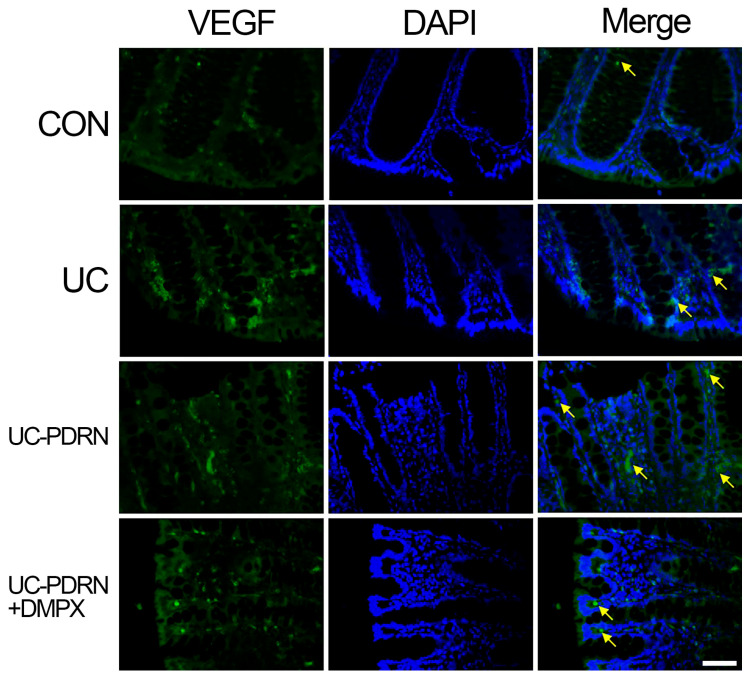
Expression of VEGF. VEGF-positive regions in merged images are indicated by yellow arrow labels. The scale bar is 150 μm. VEGF, vascular endothelia growth factor; DAPI, 4ʹ-6-diamidino-2-phenylindole; (CON) control group; (DSS) dextran sulfate sodium-treated group; (DSS-PDRN) dextran sulfate sodium and polydeoxyribonucleotide-treated group; (DSS-PDRN + DMPX) dextran sulfate sodium and polydeoxyribonucleotide with 3,7-dimethyl-1-propargylxanthine-treated group.

Figure 7 shows the effect of PDRN on VEGF expression in the colonic tissues of the DSS-induced UC mice. The expression of VEGF in UC mice was higher compared to that of the control mice. On the other hand, PDRN treatment further augmented VEGF expression beyond levels observed in untreated UC mice, indicating that PDRN may promote angiogenesis and tissue repair mechanisms through enhanced VEGF-mediated vascular remodeling. Importantly, the co-administration of PDRN with the selective A_2A_R antagonist DMPX attenuated the PDRN-induced upregulation of VEGF. This indicates that PDRN’s effect on VEGF expression is dependent on A_2A_R activation, highlighting a potential mechanistic pathway through which PDRN facilitates mucosal healing and vascular regeneration in UC.

## 4. Discussion

DSS is used as an agent that causes UC because DSS induces inflammation and damage in the intestinal mucosa [22,23]. In a DSS-induced UC mouse model, the colonic epithelium was damaged, resulting in diarrhea, weight loss, bloody stool, and inflammation, which are similar to human UC symptoms [24,25]. In the current study, we confirmed that oral administration of 3% DSS to mice for 7 days significantly reduced body weight, decreased colon weight and length, and increased DAI scores. Considering the results of previous studies, our results confirmed that UC was more severe than moderate [16,25].

Excessive production of pro-inflammatory cytokines initiates colonic mucosal damage and reduces the expression of anti-inflammatory cytokines [26,27]. In the current study, administration of DSS significantly enhanced the production of pro-inflammatory cytokines such as TNF-α, IL-1β, and IL-6, and suppressed the production of anti-inflammatory cytokines such as IL-4 and IL-10, thereby increasing the colon damage score. Therefore, modulating pro-inflammatory and anti-inflammatory cytokines becomes a therapeutic target for UC.

Inflammation progresses through several complex molecular biochemical reactions. NF-κB is a key transcription factor involved in the development of inflammation [28]. NF-κB is activated by the degradation of IκB-α through signal induction, and this NF-κB activation modulates transcription of inflammatory genes, including pro-inflammatory cytokines [29,30].

In particular, this activated NF-κB complex can translocate from the cytoplasm to the nucleus to produce transcription factors such as TNF-α, IL-1β, and IL-6 in the UC state [31,32]. In the present results, UC was caused by DSS administration, which resulted in IκB-α phosphorylation, which in turn activated NF-κB. The increase in NF-κB ultimately resulted in increased expression of pro-inflammatory cytokines and decreased expression of anti-inflammatory cytokines in colonic tissues. Current results suggest that an imbalance of inflammatory cytokines worsens the progression of ulcerative lesions of the colon. The current study showed that PDRN treatment significantly suppressed the production of pro-inflammatory cytokines and enhanced the production of anti-inflammatory cytokines.

Most cells involved in wound healing express A_2A_R, which regulates pathophysiological responses to many diseases [14,20]. A_2A_R agonists are known to be useful in the treatment of inflammatory diseases [30,33]. PDRN, an A_2A_R agonist, inhibits apoptosis and inflammation by reducing NF-κB through its expression via A_2A_R stimulation [30]. In this study, treatment with PDRN suppressed IκB-α activation and appeared to inhibit NF-κB expression in colonic tissues.

In particular, A_2A_R activation enhances intracellular cAMP level and acts as a physiological inhibitor of inflammatory neutrophil function [20,34]. A_2A_R also signals the cAMP-PKA pathway and accelerates CREB phosphorylation. This CREB phosphorylation regulates NF-κB to suppress inflammatory cytokines [35]. In the current study, PDRN treatment enhanced A_2A_R and cAMP levels in mice with DSS-induced UC. The present results suggest that enhanced cAMP expression suppressed IκB-α activation and NF-κB expression in colonic tissues. PDRN treatment was associated with activation of the cAMP/PKA/CREB pathway, which may contribute to inhibition of NF-κB signaling. These findings suggest that the therapeutic effects of PDRN may be related, at least in part, to the modulation of cAMP-mediated signaling pathways. Taken together, these results indicate that PDRN exerts its therapeutic effects through a time-dependent activation of A_2A_R-mediated signaling pathways.

VEGF is a potent angiogenic growth factor that plays a key role in promoting inflammation and modulating mucosal immune-driven angiogenesis in inflammatory bowel disease [36,37]. During UC healing, endothelial cells influenced by VEGF proliferate and form microvessels and capillary networks within the tissue [13]. Activation of A_2A_R in the setting of colonic injury stimulates VEGF production in macrophages [36,38]. PDRN accelerates the healing process by stimulating A_2A_R to promote VEGF production and fibroblast maturation [39]. The current study demonstrated that UC induction enhanced VEGF expression, and PDRN treatment further enhanced VEGF expression in colonic tissues. Given the context-dependent role of VEGF in inflammatory bowel disease, this increase may be associated with mucosal repair rather than exacerbation of inflammation. Although excessive VEGF signaling may contribute to inflammatory responses under certain pathological conditions, VEGF is also closely associated with angiogenesis and mucosal repair during tissue healing. These findings suggest that PDRN may contribute to tissue repair processes in UC, potentially through VEGF-related mechanisms. However, although the present study demonstrated the therapeutic effects of PDRN through the modulation of the A_2A_R-mediated PKA/CREB/NF-κB signaling pathway in DSS-induced ulcerative colitis, several limitations should be considered. The DSS-induced colitis model mainly reflects acute inflammatory responses and may not fully represent the chronic features of human ulcerative colitis. The present study mainly focused on signaling pathway-related mechanisms, and additional studies evaluating broader immunological and tissue-repair responses may further clarify the pharmacological actions of PDRN. In addition, long-term therapeutic outcomes were not evaluated in the current experimental setting. Further studies will help to better define the therapeutic potential of PDRN in inflammatory bowel disease.

## 5. Conclusions

PDRN treatment improved body weight, colon weight, and colon length and decreased DAI and colon histological scores in UC mice. In addition, the relationship between the specific A_2A_R antagonist DMPX and PDRN in UC models was observed, indicating that the action of PDRN was mediated by A_2A_R. Therefore, PDRN showed therapeutic effects on UC by enhancing VEGF expression and suppressing inflammation through regulating the PKA/CREB/NF-κB signaling pathway.

## Figures and Tables

**Table 1:** Wallace histological colon damage score.

Appearance	Score
Normal	0
Damage limited to surface epithelium	1
Focal ulceration limited to mucosa	2
Focal, transmural inflammation and ulceration	3
Extensive transmural ulceration and inflammation bordered by normal mucosa	4
Extensive transmural ulceration and inflammation involving entire section	5

## Data Availability

Data will be made available on request.

## Abbreviations

The following abbreviations are used in this manuscript:

UC	Ulcerative colitis
PDRN	Polydeoxyribonucleotide
A_2A_R	Adenosine A_2A_ receptor
DSS	Dextran sodium sulfate
DMPX	3,7-dimethyl-1-propargylxanthine
DAI	Disease activity index
NF-κB	Nuclear factor kappa B
PKA	Protein kinase A
IκB	NF-κB Inhibitor
cAMP	Cyclic adenosine monophosphate response element binding protein
ELISA	Enzyme-linked immunosorbent assay
TNF-α	Tumor necrosis factor-alpha
IL	Interleukin
FITC	Fluorescein isothiocyanate
